# A Scoping Review of Different Ways of Thinking in Children

**DOI:** 10.3390/bs8120115

**Published:** 2018-12-15

**Authors:** Elisa Jones Arango, Shane Costello, Christine Grové

**Affiliations:** Faculty of Education, Monash University, 19 Ancora Imparo Way, Clayton, VIC 3800, Australia; shane.costello@monash.edu (S.C.); christine.grove@monash.edu (C.G.)

**Keywords:** individual differences in thinking, children, scoping review

## Abstract

Despite the growing interest in differences in thinking, much less is known about differences in how children think and how they come to think. The aim of this scoping review is to map out the key concepts underpinning the conceptual boundaries of children’s (5–12 years of age) individual differences in thinking. The scoping review identified eight papers for analysis; all of which were set in an educational context. The findings presented inconclusive results regarding learning and thinking differences related to students’ academic achievement. This review has identified two main drawbacks with this research area. Firstly, there is little consensus between the models employed to understand the different ways children think. To further place these findings into context we look at conceptualisations of individual differences, where individuality is considered a process of stable characteristics interacting with more dynamic structures. This analysis highlights the second drawback, previous research has solely focused on exploring thinking characteristics that are not stable and are therefore subject to change depending on the context. The review found that there is little to no research which explores thinking preferences in children that are consistent across contexts and time. Moreover, there was no research identified that explored the impact of differences in thinking outside the educational domain, such as children’s wellbeing. Further research is required to identify the more stable characteristics that reflect and capture children’s different ways of thinking.

## 1. Introduction

Despite the growing interest in differences in thinking [1], much less is known about individual differences in the way children choose to think about and understand the world around them. From a development perspective, as a child grows so does their ability to think and reason. In early childhood (2–6 years of age) children no longer rely on the senses to understand their world, they gain symbolic thought, where an object or word can stand for something else. In middle to late childhood (6–12 years of age) children develop the ability to reason about concrete experience, they become more systematic, objective and scientific in their thinking [2]. These stages outline how children’s capacity to think develops to understand the world around them but these norms reflect what is typical; they describe expected characteristics of thought at a specific age of development. However, individuals show great diversity in development, such that maturation can be conceptualised as a collaboration between developmental stages and individual differences; for a review see Fischer and Silverman [3].

The study of individual differences explores how people vary across personality, cognition and thinking [4]. One of the most recognisable of these being personality, which presents a map of habitual behaviours that are stable over time and can predict important life outcomes [5]. There is mounting evidence of personality structures in children, even as young as two years of age and these have been associated to maladaptive and adaptive functioning. Research has linked personality structures to internalizing (anxiety and depressive symptomology) as well as externalizing behaviours (lack of effortful control and antisocial disruptive behaviour) [6]. On the other hand, personality in children can also predict social competence and mastery of educational and work tasks [7]. However, we are not only shaped by our behaviour, we are also largely dependent on our way of thinking. Research suggests that engrained habits in our way of thinking, predispose us towards ‘seeing’ the world in particular ways, leading us to favour an adaptive or maladaptive interpretation of an event [8,9,10]. Different ways of thinking not only impacts wellbeing but research carried out in adolescents and adults suggests that ways of thinking are integral to how individuals work together in a group and form relationships in a professional setting [11,12]. Some research suggests that different ways of thinking have predictive power for academic achievement, beyond that of general abilities [13]. So what do we know about individual differences in the way children think about the world and the impact of children’s different ways of thinking on wellbeing, social and academic domains? This scoping review hopes to shed light on these questions by mapping available research on individual differences in children’s thinking; the way a child prefers to approach a task or solve a problem. We start by reviewing the adult literature on individual differences in thinking, as there are no prominent models available that are specific to children’s ways of thinking. The grouping of various models in the field of adults provides an important context to review different ways of thinking in children.

### Differences in the Way People Think: What We Know So Far

In the late 19th century, the construct of ‘style’ was adopted by general psychology to describe differences in individual characteristics across cognitive, personality, communication and behavioural domains. Regarding the thinking domain, the term ‘cognitive style’ was employed to describe a person’s typical or habitual mode of problem solving, thinking, perceiving and remembering [14]. A surge in interest into cognitive style saw the development of a multitude of models and conceptualisations that over the years have, come in and out of style. Consequently, the field of cognitive style types is often described as handicapped or fragmented by a confusing lack of unity among a plethora of conceptualisations and operationalisations [15,16,17]. In an effort to clarify and classify the various models, authors have described three distinct approaches in style conceptualisations, centred on cognition, personality and activity [1,14]. Despite the majority of research on cognitive style conducted on adults [18,19], an understanding of the unique perspective, as well as the limitations of each approach is key to the analysis of children’s literature.

The cognition-centred approach focuses upon individual differences in cognition and perception. Models under this approach seek to understand differences in *how* information is processed by individuals, often referred to as cognitive style. Rayner and Riding [14] identified 17 different models that each describe different dimensions of information processing. These can be grouped into two broad cognitive style families: the Wholistic-Analytic family and the Verbal-Imagery family [20]. The Wholistic-Analytic family includes models that outline whether an individual prefers to process information by breaking it into parts or taking the whole view. The Verbal-Imagery family, describes preferences for thinking in words or pictures. Originating in experimental cognitive psychology, these dimensions are generally measured by performance-based tests that are binary in nature, with results ranging from one extreme to a contrasting extreme [1]. Because these are mostly tests of maximal performance, some argued that these models may tap into intellectual abilities [21]. Indeed some of these information-processing measures are closely related to ability measures [22]. A recent integration of cognitive abilities can be said to capture a wide domain of information processing and perception abilities [23]. These tests illustrate differences in children’s performance across cognitive and perception based tasks [24].

The personality-centred approach aimed to capture typical or preferred behaviour related to thinking. As opposed to viewing these as variables that resemble personality constructs, they are seen as bridging variables that embody cognition and personality simultaneously [25]. Personality-centred measures are commonly determined on questionnaire type instruments that are similar to personality inventories [1]. Popular models include the Myers Briggs Type Indicator (MBTI), which originated from Jung’s theory of psychological types [26]. The combination of four different functions: Thinking, Feeling, Sensation and Intuition, result in a total of 16 type classifications. For example, a specific type may describe a person who is practical, orderly and matter-of-fact. The MBTI is designed for adolescents (from 14 years of age) and adults. It is widely popular within organisational and occupational settings, despite some researchers finding psychometric flaws [27,28]. A similar, albeit less popular instrument, The Gregorc Style Delineator [29], aims to classify adults into different types but some suggest it has little to add when compared to the MBTI and other style measures [30]; furthermore, psychometric weaknesses are also highlighted [31]. A more recent approach is The Ways of Thinking model and instrument which describes an individual’s disposition towards thinking in predictable ways across six factors: Intuitive, Creative, Subjective, Controlled, Narrow and Sensate [17,32,33]. The model was established using a psycholexical approach [34], a method commonly employed in the field of personality research. This approach assumes that generalisations that make up everyday language result from the groupings of important individual differences. The Ways of Thinking measure showcases good psychometric properties [35].

A third approach focused on style in relation to various activities, settings and environments. The activity-centred approach dominates in the educational field, resulting in the concept of learning styles. Stenberg [36] defined learning styles as how people prefer to learn. Models under this approach can be organised into three distinct groups, each reflecting a particular dimension of the learning process [14]. Process-based models focused on how individuals perceive and process information in order to learn. Preference-based models outlined learning preferences in various dimensions (such as: light or dark environments, learning in pairs or alone). Lastly, cognitive-skills-based models mostly combined information-processing preferences with study or instructional preferences [14]. The field of learning styles has methodological, conceptual and empirical challenges, mainly due to the large number of models available, which continue to proliferate [37]. Although there is evidence for the presence of learning styles in both adults and children, the implication and practical application of learning styles for educational practice has received little support [38,39,40,41].

**Figure 1:**
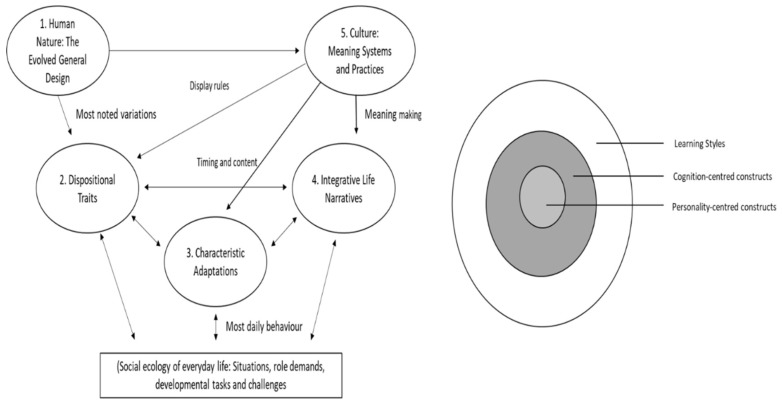
The diagram on the left represents The New Big Five model [5] Human individuality is presented as a complex interaction between five principles that represent stable to more dynamic characteristics. To the right is Curry’s Learning Style Onion [42] a framework that represents the learning process as it starts from stable structures at the core and is lastly influenced by flaky outer layers.

It is clear that each approach presents a unique perspective on different ways in which thinking can occur. But how can these distinct approaches relate to each other, if at all? To conceptualise the relationship between cognition-centred, personality-centred and activity-centred perspectives, Curry [42] proposed the Learning Style Onion framework (Figure 1). Resembling the structure of an onion, the core of the model is made up of highly stable personality and cognitive traits and outer layers are flakier, less stable characteristics that are more susceptible to change through environmental factors. By this organisation, learning is a process controlled by personality-centred constructs (core of the onion), translated through cognition-centred constructs (middle of the onion) and given a “final twist” by factors of learning styles (outer layers). Importantly, The Learning Style Onion encompasses the complementary nature of the three approaches.

Although Curry’s onion presents a metaphor grounded in the process of learning, a similar model has emerged in the personality field. The New Big Five model is a framework for understanding individuality through an integrative view of the whole person [5] (Figure 1). Five principles present different components of human individuality and like Curry’s onion they are organised from stable to more variable and dynamic structures. The core of the New Big Five model, principle 1, is human nature: species-typical characteristics that drive us to survive and reproduce. Principle 2 are personality traits in the Big Five-Factor model [43] which outline a set of behaviours that are stable throughout adulthood [44,45]. This is followed by principle 3 where aspects of human individuality speak to motivational, social-cognitive and developmental variables that are contextualised in time, situations and social roles. Principle 4 presents live narratives, evolving life stories that establish identity and integrate individuals into modern life. Finally, principle 5 sets the stage to which individuals attune their characteristic design: culture and the environmental arrangements of everyday life.

McAdams and Pals [5] assert that to make sense of the various and diverse approaches to personality research, one must take into account all the principles that together interact to shape the whole person. An integration that the field of cognition/thinking/learning styles needs but often fails to be acknowledged [19]. So to make sense of the multitude of approaches to differences in thinking, one may consider that the process of thinking is shaped by various layers. Describing differences in the way people think, involves more than only understanding different dimensions of information processing. Individuals have contrasting personalities that differentially influence their modes of cognition and behavioural expression [25]. The context, environmental and cultural factors, will also play a role in the way an individual expresses and appropriates their thinking. Different ways of thinking can then be viewed as a conflation of personality, cognition and environmental factors. Both Curry’s Learning Style Onion and The New Big Five model present as useful frameworks for conceptualising the process of thinking and how this can be influenced by different layers. Literature focused on adults mostly failed to integrate or acknowledge the different conceptualisations [19] but the extent to which this occurs in children’s literature is not known.

The study of differences in cognitive style has extended from adults to adolescents (mainly through an exploration of learning styles in secondary and tertiary education [18]), however there appears to be less research pertaining to individual differences in thinking in children. Therefore, the current review aims to summarize the literature available on children (5–12 years of age). Investigating individual differences in this age group will provide insight into if or how the preferences in thinking of children may differ from adolescence and adulthood. This comparison can offer a unique perspective to the pathways of individual growth. For example, the study of individual differences in temperament and personality structures in children has been linked to recent advances in our knowledge of genes and neural networks [46], differences in social interaction [47] and psychopathology [48,49]. These are complex interactions [6] and therefore insight into individual differences in children’s thinking may shed light on development of behaviours and life outcomes that are yet to be captured through a personality spotlight [50]. Understanding the models currently employed to capture different ways of thinking in children will also clarify directions for future research. To discuss current research on differences in children’s thinking, this scoping review will use the three conceptualisation approaches (cognition-centred, personality-centred and activity-centred) employed in research with adults. To summarise the literature, both Curry’s Learning Style [42] Onion and The New Big Five model [5] are employed.

## 2. Methods

Scoping reviews can be used to map the key concepts underpinning a research area as well as to clarify working definitions, and/or the conceptual boundaries of a topic [51]. The objective of this scoping review is to examine and map the range of literature available on individual differences in children’s thinking. Scoping study research questions are broad in nature as they tend to focus on summarising breadth and depth of evidence [52]. As such, this review will aim to: (1) summarise findings presented by the papers, (2) classify the style models currently employed in research of different ways of thinking in children, according to: cognition-centred, personality-centred and activity-centred conceptualisations, (3) identify gaps in the current literature, by employing Curry’s Learning Style Onion [42] and The New Big Five model [5] as over-arching frameworks.

### Procedure

The problem was defined as a need to examine and map the range of literature that comprises differences in thinking in children. By the age of 12 children are able to perform logical operations and can reflect about their experience [2]. To focus research on children, studies which reported the age of participants to range from 5–12 years, were included for analysis. Previous reviews that focused on adult populations [18] were used to identify key words used in the field, which included: ‘cognitive style/,’ ‘thinking style/’ or ‘learning style/.’ These key words were inputted into ProQuest, PsycINFO and ERIC e-journal library separated by the phrase ‘or’ and searched for anywhere in the article during May 2018.

Inclusion criteria comprised of the age (5–12 years) of the study population, English language and full text peer-reviewed journal articles or masters and doctoral theses focused on thinking/cognitive/learning style in children. This was conceptualised as a preferred way for children to understand or think about what is going on around them. Articles from all parts of the world, as well as from different fields outside psychology, such as education, were included in the analysis.

Duplicate articles and non-English articles were excluded from analysis. With an emphasis on capturing more recent developments in the field, articles that were published before 1998 (exceed 20 years since publication) were excluded. Articles were also excluded if they did not address preferred ways to think in children. The identified articles (n = 1921) were grouped into various categories (see Appendix A). Articles that pertained to children’s learning style, as well as teaching style, were included for review with an emphasis on gathering information provided about children. However, those only pertaining to teaching style were excluded from review. This is due to the focus of the current study on the individual’s thinking preferences, not the way the information is delivered to the individual. Articles were excluded that related to the development of children’s thinking, because this review does not concern the capacity or constraints placed on thinking or understanding at different stages of development. On a similar basis, work focused solely on metacognition was also excluded. A large number of studies were placed under “miscellaneous” category as they did not conform to a specific group. For example, the articles related to music, sleep or judicial decision making in children. Other categories pertained to clinical populations (example: “The development of route learning in Down Syndrome, Williams Syndrome and typical development”); papers that outline learning interventions (example: “Watching, creating and achieving: Creative technologies as a conduit for learning in the early years”) or addressed the language literature (example “Children’s learning strategies in language immersion classrooms”).

After initial review of abstracts and full articles seven were excluded because they solely addressed teaching style, 11 were pertinent to adolescent or adult populations and six were irrelevant. Four articles were reported to occur in a primary education setting but upon further investigation of the age of participants it was revealed students ranged from 13–14 years of age and were therefore excluded from analysis. Two additional articles were identified through a reference search, yielding a final number of 8 articles for analysis. The age of participants for all eight studies ranged from 8–12 years. The investigator read each article and completed a study details form. This included the article source (location and date), study methodology (study design, population and theoretical model) and study findings (See Appendix B). Two researchers’ independently coded and then compared their responses to the completed study summary form [52]. Specifically, identification of the main findings section yielded a 100% inter-rater agreement level.

## 3. Results and Discussion

### 3.1. Findings from the Research

All of the eight studies were set in an educational setting and the majority aimed to investigate the relationship between learning styles and academic achievement. The results are presented by grouping the studies according to their aim: Three papers focused on learning styles and academic achievement, three papers explored the role of matching students’ learning styles to the classroom practices, one paper compared students’ perceived with reported learning preferences and one paper developed their own learning style measure. 

Three papers explored the relationship between learning styles and specific academic domains. Firstly, Nicolaou and Xistouri [53] employed the field-dependent/independent cognitive style measure [54], to explore students’ abilities to pose mathematical problems. This style measure suggests that field-independent individuals tend to break information apart and field-dependent individuals focus their attention globally [54,55]. The authors reported that field-independent students had the highest ability to pose mathematical problems. They suggested that field-independent students possessed more analytic abilities, which in turn facilitated problem construction. Secondly, Baltaci, Yildiz and Özcakir [56] employed the Marmara Learning Styles Inventory, which specified students’ environmental preferences for learning. It was reported that students with the highest academic achievement in mathematics reported a preference for auditory learning. No reasons were provided to explore why this could be the case. Finally, Chaiyapornpattana and Wongwanich [57] explored the relationship between students’ thinking style, classified by the authors’ own scale and general academic achievement. Of interest, it was found that, most girls affiliated with a preference for working alone (internal style), whereas most boys preferred tasks that allowed them to work in groups (external style). The authors suggested that this may be a reflection of Thai culture and upbringing, as boys are exclusively encouraged to showcase leadership and work in groups. Regarding academic achievement, students with judicial (like to evaluate rules and procedures), hierarchical (likes to prioritise goals) and liberal (individuals who can tolerate change to the way things are normally done) thinking styles attained higher academic results across the board. The authors suggested that the characteristics of time management and prioritising assisted students in attaining higher academic grades. In summary, each paper presented their own set of preferences and styles that appear to be advantageous for a specific academic domain or general achievement. As such, it is difficult to draw firm conclusions about the relationship between learning or thinking styles and academic achievement.

Three papers explored the effect of matching student’s learning preferences to their environment. A student’s learning preferences are identified through an inventory. A “match” occurs when the preferences identified by the student (for example, a preference for information presented visually) is in accordance with the classroom environment (the teacher supplements oral information with visual information). Wilson [58] compared academic achievement between students whose learning styles, measured by the Computerized Assessment Program-Styles of Learning (CAPSOL), matched the classroom practices, with students whose learning styles did not match their classroom practices. Importantly, the classroom practices were not manipulated to match or mismatch students’ learning styles, rather the groups were identified as such from the existing classroom structures. The findings outlined that students whose learning style matched their classroom practices had similar academic results to their non-matched counterparts. This finding suggests that academic achievement is not dependent on reported learning preferences. On the other hand, Peklaj [59] examined the effect of a co-operative learning environment on academic achievement, according to students’ cognitive style. In this instance the teaching practices were manipulated, such that some students received instruction based on the principles of co-operative learning, where emphasis is placed on group work. Whilst another group of students continued to receive the conventional teaching method. Students were classified to have either field-dependent or field-independent cognitive style. Field-dependent individuals have a preference for working in groups, whereas field-independent individuals prefer to work independently [55]. The study reported that co-operative learning was most effective for students who had a preference for working in groups (field-dependent). Field-dependent students displayed the highest gain in mathematics and language achievement tests. More recently, a study employed the same methodology but reported different results [60]. The authors reported that co-operative learning increased post-test results in mathematics, irrespective of whether students had a preference for working independently (field-independent style) or in groups (field-dependent style). However, in the language domain, co-operative learning was only effective for individuals who identified a preference for working independently (field-independent). In summary, the studies present mixed results on whether the teaching practices should aim to match student’s learning style [58,59,60]. Moreover, because there is a focus on the environment and teaching practices, these studies speak less about how differences are manifested in children’s thinking.

Boström [61] compared students’ learning style, classified by the Dunn and Dunn Learning Style Inventory (LSI), to their perceived learning preferences, captured through a group discussion. It was highlighted students perceived and expressed a higher degree of learning preferences then was evident through their learning style inventory. For example, out of 40 students, 37 reported a preference for learning in the afternoon through group discussion; when the LSI revealed that only 13 students indicated this preference. However, as the authors highlighted, social desirability may have largely influenced responses. Individual interviews revealed that children (10 years old) have difficulty explaining when they know they have learned something new. The questions included in this interview all addressed preferences for learning however and the topic of different ways of thinking was not addressed. The paper highlights the need to explore differences in the way children think by gaining a sense of the language and concepts children themselves use to explain their own ways of thinking.

Finally, Güven and Özbek [62] developed their own scale to determine the learning style of primary education students. The result is a 27-item questionnaire that presents 8 factors, with reliability and validity reported by the study but no external evaluation available. Most factors addressed environmental aspects of learning, such as the level of noise of the environment, visual aids required, the need to move when studying and the degree of writing preferences for learning. As it stands, the measure is simply adding to the proliferation of activity-centred models (learning styles) [18].

So what do these studies tell us about children’s thinking? At best, these results highlight that children at this age (8–12 years) presented with individual differences in the way they preferred to learn and think. Whilst some preferences for learning and thinking were identified by some studies [53,56,57], there appeared to be little consensus between them. It was also difficult to conclusively determine the effect of these individual differences on student’s academic achievement [58,59,60]. A major drawback from the research identified is the fact there is little consensus on the models employed, so studies relied on varying theoretical perspectives and captured different constructs. 

The next section discusses the effect of employing so many different models on our understanding of children’s differences in thinking. It is also important to consider the nature of the constructs that these models aim to measure, to gain an understanding of the scope and limitations of each measure. To do so, we employ Curry’s Learning Style Onion [42] and The New Big Five model [5]. As the purpose of this next section is to provide a brief summary of the various models that were employed, refer to Coffield and colleagues [18] for a thorough review and critique of the more prominent models.

### 3.2. Classifying Style Conceptualisations and Identifying the Gaps 

#### 3.2.1. Activity-Centred Models 

Five of the papers available for review adopted an activity-centred approach, with five individual models identified in total. These models all explored learning styles but each addressed a specific dimension of the learning process [14]. Three models focused on students’ learning preferences across different dimensions (preference-based). One of these was a widely used model, the Dunn and Dunn Learning Styles [63], which explores learning preferences across environmental, emotional, physical and sociological dimensions. Mainly, this model outlines factors that can be manipulated, like the time of day, learning in groups or individually and preferences for visual, auditory or kinaesthetic/tactile learning. However external reviewers dispute the validity and reliability of the model [64]. The other models that fall under the preference-based approach [56,62], measure one, or multiple, dimensions presented by Dunn and Dunn Learning Styles. One of these models was developed and tested by the authors [62], with no external evaluation of validity or reliability available. Finally, the Marmara Learning Style Inventory [56] was developed as part of a master’s thesis that was not available for review.

Two papers employ models that fall under the cognitive-skills-based category of learning styles: the CAPSOL [65,66] and Stenberg’s Thinking Styles Inventory (TSI) [67]. The CAPSOL samples a combination of preferred learning environments and dimensions (visual/auditory/tactile learning and group versus individual work); as well as preference for information processing, based on ordering and prioritizing (sequential) or understanding the big picture (global). This measure is reported to have good face validity, as well as good test-retest reliability [58] but we were unable to identify the source of this claim through reference check. The second model is Sternberg’s Thinking Styles Inventory. The TSI was derived from the theory of mental-self-government, which presents a metaphorical organisation of thinking that resembles governmental structures. The model presents 13 different learning styles; for each preferred method of instruction and assessment are recommended. External reviews hold reservations about the model and underlying theory. The theory outlines 13 different thinking styles, deducted from vague and simplistic statements but moreover the main critic lies in the practical implication of catering to 13 different styles in one classroom [18] (pp. 114–117).

The models discussed above were all employed in the present studies to explore different learning styles in children, suggesting that through these questionnaires children were able to report learning preferences. However, if we consider learning to be a process, as outlined by the Learning Style Onion, the activity-centred approach translates to the outer flaky layers. By this organisation these instruments measure dimensions that are prone to change and thus less instrumental to the learning process. If learning preferences identifying through these models were stable it would follow that presenting a learning environment that matches students’ learning preferences would be advantageous, known as the matching hypothesis. However, previous studies have concluded a lack of evidence for modality preference (auditory, visual or tactile modes of presenting information) as a guide to teaching style [68]. This was consistent with the conclusion reached by Wilson [58] in her study that focused on children.

The outer layers of Curry’s onion are analogous to Principle 5 in The New Big Five model. This level explored behaviour as a product of the complex interaction between person and environment, in particular their social and cultural contexts. At this level, values and expectations play a pivotal role as boundaries by which people express their traits [5]. Here the context means more than just the setting in which learning takes place. This is highlighted by the findings of Chaiyapornpattana and Wongwanich [57], as the thinking styles of boys and girls reflected important gender roles reinforced in Thai upbringing; boys held a preference for working in groups whilst girls preferred to work independently. Considering that culture is an important context that may influence the way in which thinking and learn are expressed, it is important to note that this review identified papers from eight different cultures. Therefore, the different cultural contexts present in each study may lead to different conclusions regarding the distribution of learning preferences. However the studies identified did not address cross cultural comparisons. 

The activity-centred models employed by the studies may have reported children’s learning preferences but the variables captured through these instruments are fluid and prone to change depending on the context. When aiming to identify learning styles in children, future research should be mindful of the psychometric properties of the model they employ but most importantly consider the variable nature of preferences identified through the activity-centred approach and as such the implications that arise for practical application. 

#### 3.2.2. Cognition-Centred Model.

Three papers employed Witkin’s field dependent/independent model [54], falling under a cognition-centred approach. From this perspective, preferred ways to process information are established between two poles using the Embedded Figure Test (EFT): field dependent and field independent individuals. Although the theory initially focused on perception, specifically the ability to ‘dis-embed’ a shape from its surrounding field, it was later extended to explore functions that applied to teaching and learning [69,70]. Field-independent individuals are said to prefer independent activity, structure their own learning, have self-defined goals and respond to intrinsic reinforcement. At the other end, field-dependent describes individuals who have a preference for learning in groups, need higher levels of extrinsic reinforcement/direction and prefer an established structure.

Witkin’s field-dependent/independent model translates to the middle layer of Curry’s Learning Style Onion, the individual’s intellectual approach to assimilating information. The structures measured at this layer appear to be more stable and are closely related to ability measures [21,22]. However, the Embedded Figures Tests classifies individuals into two opposing poles and it is worth noting that a large group of individuals do not fall into either category [59,70]. This cognitive and perception layer can perhaps be captured fully by ability measures that provide a relative standing across a spectrum, as opposed to a binary outcome [23].

In summary, the eight papers employed six different models, with little reasoning or explanation provided for why each model was chosen. Some argued for choice on the ground of “most studied” or “popular” [60,61,62] and provide little reference to the psychometric properties of the model employed (or the potential psychometric flaws in most cases). It is evident that there is considerable similarity between the models within each conceptualisation. That is, models that focus on learning preferences across dimensions all address one, or more, of these dimensions (environmental, emotional, physical and sociological); some are more comprehensive than others. It can be argued that there is considerable overlap between the three conceptualisations, with some activity-centred models including cognition-centred components, for example Dunn and Dunn Learning Inventory [63] and Stenberg’s Thinking Styles measure [67]. Over two decades ago, Messick [25] (p. 131) described the cognitive style literature to ‘seem either vague in glossing over inconsistencies or confused in stressing differentiated features selectively.’ The current scoping review identified research that is relatively recent (oldest article is from 2003) but it appears that little has changed as this review of literature still upholds similar, if not the same problems. Some models are narrow in scope and fail to account or address the whole process of thinking and often provide limited psychometric properties of instruments.

This scoping of the literature on different ways children think largely resembles the adult literature, which is predominantly dominated by activity-centred models, followed by cognition-centred models. The gap is clear. Aside from there being a limited number of papers focused on children, there were no research that employed personality-centred models, or that speak to more stable thinking preferences - the core of Curry’s Learning Style Onion. This is not exclusive to the current review, with Coffield and colleagues [18] concluding that ideas about stability are heavily influenced by theoretical orientation, instead of empirical evidence. If we consider The New Big Five model, what lies at the centre are personality traits, habitual behaviours that are stable over time and across contexts [5]. Are there equivalent ‘thinking’ traits? To the authors’ knowledge the only empirically validated measure of personality-centred thinking traits available for use in adult populations is the Ways of Thinking model [17], which is yet to be tested in children. 

## 4. Conclusions

The scoping review identified eight papers that addressed how children think about or understand the world around them. All studies were set in an educational domain and predominantly explored the relationship between learning styles and academic achievement. It was identified however that each paper presented its own set of findings, with little consistency between findings to draw any firm conclusions about *what* the differences in children’s ways of thinking actually are. At best, we can conclude that individual differences in thinking are likely to be present from a young age (8–12 years of age). From the eight studies available for review, six different models were employed to understand differences in thinking and learning. The variability of results can be partly explained by the wide variety of models employed, each with its own unique set of theoretical underpinnings. Furthermore, many of these models lack psychometric rigour, which undermines the findings presented by some of the papers. Understanding individual differences is complex because individuality is a dynamic process that combines many different layers. Curry’s Learning Style Onion and The New Big Five model present how these different structures, that are formed from stable to more variable characteristics, come together to create the process of learning, in the former and the expression of the “whole person.” This review highlights how the majority of available papers pertain to explore the more variable characteristics of thinking in children. Importantly, there was no research identified that targeted more stable thinking traits. Furthermore, the studies identified were all set in an educational context and fail to acknowledge other aspects of children’s lives: well-being, relationship formations or important life outcomes. This review clearly highlights that there is a long way to go in understanding individual differences in the way children think and understand the world around them.

## Figures and Tables

**Figure A1:**
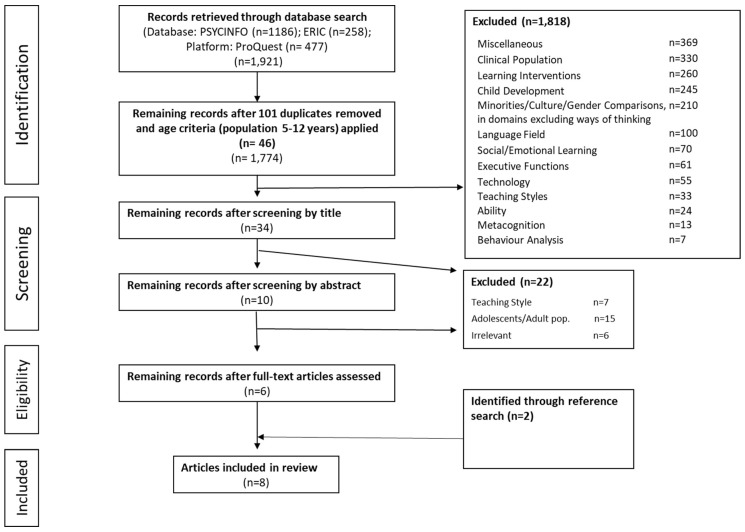
Flow Chart of Study Identification.

## Appendix B

**Table A1:** Key Summary of Findings.

Authors	Population	Conceptual Model	Purpose(s)	Findings/Implications
Peklaj (2003) [59]	Primary School students (11–12 years, n = 373, Male and Female) *Slovenia*	Field-dependence/independence *Cognition-Centred*	Comparing co-operative to individualistic learning environment to identify differences in student’s achievement according to their cognitive style, field-dependence/independence.	1. Co-operative learning lead to increased academic attainment in maths and language domains 2. Field-dependent student’s showed the greatest improvement in co-operative learning classrooms in both math and language subjetcs
Güven and Özbek (2007) [62]	Fifth Class Primary School (no age specified, n = 211, Male and Female) *Turkey*	Development of own scale *Activity-Centred (Preferenced-based)*	‘The aim of the research is to improve learning style inventory which can be used to determine the learning features of students who study in the fifth class elementary school (Turkey)’	1. 27 question inventory, with 8 Factors (Reliability Cronbach alpha: 0.78)
Nicolaou and Xistouri (2011) [53]	Sixth Grade Students (11–12 years of age, n = 94, Male and Female) *Cyprus*	Field-dependence/independence *Cognition-Centred*	‘To explore the relationship between Field-dependence/independence (FDI) and mathematical problem posing ability’	1. Field-independent participants outperformed field- mixed and field-dependent students on both ability to problem pose and complexity of problem posing 2. The context of the problem informal (with picture) compared to formal (no picture) influenced task performance, whereby field-independent students had the best performance in the informal task but there was no difference between groups in the formal task.
Wilson (2012) [58]	Fourth Grade Students (n = 200, Male and Female) *United States of America*	Computerized Assessment Program Style of Learning (CAPSOL) *Activity-Centred (Cognitive-skills-based)*	‘To identify the extent to which learning styles influence the educational process as well as academic achievement’	1. There is no relationship between matching learning styles and achievement in areas of English, Math, Arts and Social Studies
Boström (2012) [61]	10-year olds (n = 71, Male and Female) *Sweden*	Dunn’s Learning Style Inventory *Activity-Centred (Preference-based)*	‘To compare how student think that they learn to their learning styles preferences according to Learning Styles Inventory’	1. Students estimated more learning preferences then was evident through the Learning Styles inventory assessment 2. Interviews showed that students have difficulty explaining how they learn 3. Responses on learning showed that they were building their insights on the school context and experience
Chaiyapornpattana and Wongwanich (2013) [57]	11–12 years old (n=1545, male and female) *Thailand*	Development of own scale based on Theory of Mental Self-government *Activity-Centred (Cognitive-skills-based)*	‘To develop a multidimensional thinking styles scale based on theory of mental self-government for sixth grade students’	1. Questionnaire with 5 dimensions: function, form, level, scope and learning 2. Gender was correlated to dimensions 3. Academic achievement was correlated to dimensions
Vega and Hederich (2015) [60]	8–12 years old (n = 76, Male and Female) *Colombia*	Field-dependence/independence *Cognition-Centred*	‘This study is expected to determine the impact of a program based on cooperative learning methodology and the differential impact according to cognitive style’	1. The three cognitive style groups were positively affected from the co-operative learning situation in mathematics but not in the language domain
Baltaci, Yildiz and Özcakir (2016) [56]	10–11 years olds (n = 330, Male and Female) *Turkey*	Marmara Learning Style Inventory *Activity-Centred (Preference-based)*	‘Examine the relationship between metacognitive differences, learning style, genders and mathematics grades’	1. Learning styles are associated to achievement in mathematics 2. There is a relationship between Metacognitive awareness and learning styles
